# A Meta-Analysis on the Effects of Therapeutic Recreation Programs for the Elderly

**DOI:** 10.3390/ijerph17207367

**Published:** 2020-10-09

**Authors:** Eui Jae Kim, Hyun Wook Kang, Seong Man Park

**Affiliations:** 1 College of Sport Science, Dankook University, Cheonan-si 31116, Korea; 4770423@hanmail.net; 2 College of Liberal Arts, Dankook University, Cheonan-si 31116, Korea

**Keywords:** the elderly, therapeutic recreation program, meta-analysis, effect size

## Abstract

This study gathered previous research on the effects of therapeutic recreation programs for the elderly conducted in Korea in order to investigate the average effect size as well as the factors that influence the effect sizes. This study connoted findings of individual studies related to therapeutic recreation programs for the elderly from 2000 to 2018. A total of 15 papers were selected for meta-analysis. To analyze the data, a comprehensive meta-analysis 2.0 software program was used. Firstly, the overall mean effect size of the therapeutic recreation programs for the elderly was 0.644, and it was medium size. Secondly, for each dependent variable, the mean effect size on social emotional domain was 0.739, the mean effect size on physical domain was 0.548, and the mean effect size on cognitive domain was 0.485. Thirdly, major factors that influence the effect of therapeutic recreation programs for the elderly appeared to be the program period and hours per session. The results of this study prove that therapeutic recreation programs for the elderly can be an effective way to bring along a positive change, and show that the program period and hours per session are crucial factors in the design of therapeutic recreation programs for the elderly.

## 1. Introduction

In today’s world, aging has become a global occurrence. In the case of South Korea, the proportion of the elderly who are 65 years or older increased to 7% in 2000, meaning that Korea was labeled as an aging society. Furthermore, starting in 2017, the proportion of the elderly again increased even higher to upwards of 14% or more, which led to the categorization of an aged society [1]. This rapid increase in the percentage of the Korean elderly, along with the overall aging of the general population, has led to many social problems; thus the lives of those of older age are becoming a major concern in our society. 

The period of aging is one where many physical, psychological, and social changes will occur. In particular, during this period, a great decline in physical function due to natural biological aging compounded with their higher susceptibility to diseases compared to younger age groups is the reality for many older individuals [2]. For example, according to the data on the prevalence of dementia amongst the elderly [3], 88% of the elderly in Korea were reported to have one or more chronic diseases. An increase in the chronic disease rate will lead to a burden in medical expenses for the elderly individual, but will also result in a major financial dent to society and the economy as a whole. Therefore, efforts in improving the general health for the elderly is needed.

As a means of promoting the health of the elderly, leisure recreational activities have an important role. These activities not only help the elderly in maintaining their physical and mental health, but also allow them to satisfy their individual needs and help them integrate into their own communities and improve the quality of their everyday lives as a whole by allowing them to form close and meaningful relationships [4]. 

In the case of developed countries, leisure recreational activities have been used for therapeutic purposes, resulting in a major reduction in problems for the elderly [5]. Therapeutic recreation refers to the process of using recreational activities to change individuals’ physical, emotional and social behavior towards a more desirable direction [6]. Therapeutic recreation, whose types include physical activities, music, dance, arts and crafts, social activities, and nature and outdoor activities, is used as one of the means for successful and active aging for the elderly [7]. In addition, therapeutic recreation programs and leisure recreational activities are different in that therapeutic recreation is a special application of recreation that intervenes with a specific purpose and has a systematic philosophy, method, and process [8]. 

Similarly, many cases in Korea have discussed the effectiveness and application of therapeutic recreation programs for the elderly. Looking at previous studies that have validated the effects of therapeutic recreation programs in the elderly, it was reported that treatment and recreational programs were effective in the improvement of elderly individuals’ social, physical, emotional, and even their cognitive domains [9,10,11,12,13,14,15]. These results clearly show that therapeutic recreational activities have a significant effect on the improvement of health in the elderly.

However, based on other individual studies, it is necessary to recognize that the generalization of therapeutic recreation programs and their effects on the elderly are limited. According to previous studies, the program effectiveness is based on statistical significance. However, statistical significance is affected by the size of the sample and has properties of stochastic information [16], so there is a limitation in the specific and practical information of its effectiveness. Based on this perspective, it is necessary to verify the practical significance of the recreation treatment program in the elderly in order to determine its effectiveness.

Furthermore, if you look at the previous studies, you can see that the characteristics of the participants, the composition and content of the programs, the types of dependent variables, and the design of the study are different from one another. With this difference in mind, it can be predicted that the effectiveness of the program may differ depending on the research characteristics of individual studies. Within this context, there is a need for research that will aid in identifying the factors affecting therapeutic recreation programs and its effects on the elderly.

In this study, it was decided that a meta-analysis would be needed to examine the actual effects of therapeutic recreation programs and to determine what factors are causing the deviations in the programs. Meta-analysis is a comprehensive analysis method that systematically and quantitatively analyzes various research results on the same topic [17]. Meta-analysis can overcome the limitations of individual and existing literature review studies, and systematically analyze individual studies to draw comprehensive conclusions [18]. Due to this characteristic of meta-analysis, many attempts to comprehensively analyze research materials in various academic fields have been increasing, and meta-analysis research is being actively conducted in the field of physical education as well.

In view of the above, this study intends to conduct a meta-analysis on research materials that have verified the effectiveness of therapeutic recreation programs on the elderly. Specifically, the purpose of this study is to use meta-analysis to determine the efficiency of the treatment and to search for the meaningful variables that control the effectiveness of the program. There are expectations that this attempt will provide a useful resource for the establishment of follow-up research related to the therapeutic recreation effects of the elderly while developing and building a therapeutic recreation program for the elderly in education. 

## 2. Research Method

In this study, the following five-step procedures of meta-analysis were referenced to conduct the research [17]. 

### 2.1. Research Topic Selection and Research Questions

In order to achieve the research purpose of this study, the following research questions were put into place. First, how big is the overall effect of the therapeutic recreation programs for the elderly? Second, what are the variables (regulatory variables) that affect the effects of the therapeutic recreation for the elderly? 

### 2.2. Literature Search and Selection

In this study, in order to conduct research on the effectiveness of the therapeutic recreation program for the elderly, multiple sources of data coming from dissertations and journal articles published in Korea between 2000 and 2018 were collected. The criteria to which a paper would be chosen for the meta-analysis based on the collected data were as follows; first, a study that applied a therapeutic recreation program on the elderly whose standard is set at over 65 years old based on the Korean Domestic Welfare of Senior Citizens [19]; second, an experimental study of the therapeutic recreation program on the elderly; third, in the case of experimental study, a one-group pre-test–post-test design study; fourth, a study that displays statistic values (average, standard deviation, etc.) that can calculate the effect size.

### 2.3. Collection and Selection of Data to be Analyzed

For the collection of data to be analyzed, online DB were used, such as Korean Academic Information and Research Service [20], Nuri Media DB-PIA [21], Korea Academic Information Service [22]. The search terms were set to “elderly” and “therapeutic recreation” and all related literature to the topic of the study was first collected. Then, the final selection of the data for the analysis among a total of the 484 studies was conducted as follows: first, after excluding 341 duplicate papers, the remaining 143 papers were selected; second, 47 papers that were non experimental studies were excluded through the review of titles and abstracts; third, 75 studies where the research participants were not the elderly and 5 studies where the study design was a not a single group pre-post design were again excluded through the review of the entirety of each paper. Through this process, a total of 15 final studies was selected as the targets for the meta-analysis (Table 1). 

### 2.4. Coding

The final information required to calculate the effect size from the research selected as the final meta-analysis target was recorded in the coding form. The coding standard was selected so that the information presented by the research subject to be analyzed was not lost. First, the coding format included the author, the year of publication, the title of the article, and the type of publication. Second, the participants’ characteristics, characteristics of the programs, and dependent variable information were included. Third, information required to calculate the effect size (average, standard deviation, etc.) was included. Table 2 shows the summarized coding form. It should be noted here that, in order to secure the reliability of coding, all the coding courses were conducted following the opinions of two Ph.D. holders in Physical Education (one in leisure recreation and one in physical measurement evaluation) in trying to solve the discrepancy through this prior consultation.

### 2.5. Data Analysis 

The EXCEL Program (Dankook University, Cheonan, Republic of Korea) was used as the analysis tool for data coding, and the Comprehensive Meta-Analysis Program was used to analyze the effect size, homogeneity test, and publishing bias. 

#### 2.5.1. Effect Size Calculation and Interpretation

To calculate the effect size, a standardized average difference (Cohen’s d) calculation formula was used to divide the difference between a single group’s posterior and prior average by the integrated standard deviation. Based on prior research, in the case of the standardized average difference in effect size, if the number of cases is small, the effect size tends to be overestimated. Therefore, in this study, a process of converting the convenience of a small sample to a corrected effect size (Hedges’) was performed using the formula proposed by Hedges and Olkin [23].

(1) $$g=J\;\times d\left(J:correction\;facor\right)\;J\;=\left[1-\frac{3}{4\left(n_{1}+n_{2}\right)-9}\right]\;or\;\left(1-\frac{3}{4df-1}\right)$$

J = Calibration Index; d = Effect Size.

As a method of interpreting the calculated effect size, Cohen [24] suggested the effect size analysis criteria as follows: if the effect size is 0.30 or less, it is interpreted as small, 0.40 to 0.70 as medium, and the large size would be of 0.80 or more, which can be viewed in Table 3. In addition, another method of analyzing the effect size was used, where the effect size was analyzed by comparing the experimental group and the control group in the normal distribution curve by converting the effect size to the Percentiles of non-overlap. 

#### 2.5.2. Model Selection for Analysis

In meta-analysis, the model for analysis is divided into a fixed effect model and a random effect model. Depending on which of the two models is selected, the estimate and precision of the average effect size will vary [16]. In this study, an analytical model was selected from the homogeneity test statistics (Q test) and heterogeneity statistics (I2 test). The statistics used in the test of homogeneity refer to the observed variance of the effect size and the formula for calculating the statistics was as follows:

(2) $$Q\;=\sum w_{i}{\left(d-\bar{d}\right)}^{2}=\sum {\left(\frac{d-\bar{d}}{S}\right)}^{2}$$

Q: Observed Variance (Total Variance); W: Assigned Weight; d: Effect Size.

In addition, the statistical value indicating the heterogeneity of the effect size means the ratio of the actual variance, and the calculation formula of the statistical value is as follows:

(3) $$I^{2}=\frac{Q-df}{Q}\times 100\%$$

I2: Percentage of variance in a meta-analysis; Q: Observed Variance (Total Variance); df: Degree of Freedom.

Generally, 25% is interpreted as small, 50% is medium and 75% is to be considered very heterogenous (Table 4). 

#### 2.5.3. Moderator Analysis

It is necessary to identify the cause of heterogeneity if the effect size calculated in the study subject for meta-analysis is heterogeneous through the homogeneity test and the heterogeneity statistics. Therefore, it is possible to grasp a variable that causes a difference in the average effect size. In the study, a control effect analysis was conducted by performing subgroup analysis and meta-regression analysis, according to the attributes of the variables suggested in the target study.

#### 2.5.4. Publication Bias

Publication bias means that the research showing positive findings in meta-analytical research takes up a larger proportion, resulting in bias, which in turn may cause distortion in the meta-analytical results [25]. Therefore, it is important to be careful in the interpretation of the meta-analysis results when there is a publication bias. In this study, Funnel plots and Trim and fill were used to avoid the publication bias. First, it was determined whether there was a visual deflection through a funnel plot, and then the asymmetry was corrected through a Trim and fill method developed by Duval and Tweedie [26].

#### 2.5.5. Independence Assumption and Analysis Unit Shift

Meta-analysis assumes the independence of the research to be analyzed. However, if you look at the results of the research, you may have problems with the assumption of independence because there are cases that report multiple results. Therefore, in this study, the effect size was analyzed by setting the individual study as an analysis unit when estimating the overall average effect size [27].

## 3. Results

### 3.1. Characteristics of the Research to be Analyzed

In order to understand the characteristics of the selected research for meta-analysis, technical statistics were analyzed by dividing the research status by year, publication type, research object, gender, number of participants in the program, duration, number of sessions per week, time per session, and type of dependent variables. However, participants’ family history or cultural background could not be considered in the study since most of the research did not provide information on it. The characteristics of the research subject to be analyzed are shown in (Table 5). The research statuses by year are 12 (79.9%) from 2000 to 2009 and 3 (20.1%) after 2010, while the publication types are 10 theses (66.7%) and 5 journal articles (33.3%). With regard to the research participants, there were six of the elderly who were healthy (40.0%) and nine of the elderly (60.0%) who were diagnosed with some form of disease. The genders of the research participants consisted of seven female groups (46.7%), seven mixed gender groups (46.7%), and one unlabeled group (6.7%). As a result of examining how the gender variable affects the effect size, the effect size was found to be relatively higher in the female groups, but the difference was not significant. Thus, it can be assumed that studies involving only women may not significantly affect the effect size. The number of participants in the program was 10 studies of 10 or fewer (66.7%), 3 studies of 11 to 20 (20.0%), and 2 studies with 21 or more (13.4%). The duration of the programs included 2 for 6 weeks (13.3%), 3 for 6 weeks (20.0%), 1 for 9 weeks (6.7%), 4 for 12 weeks (26.7%), 1 for 16 weeks (6.7%), 1 for 17 weeks (6.7%), and 2 unmarked (13.3%). Concerning the frequency of application of the program, there were six studies once a week (40.0%), seven twice a week (46.7%), and two unmarked (13.3%). The time per program session consisted of 11 for 60 min or less (73.3%), 3 for over 60 min (20.0%), and 1 for non-indicated (6.7%). In regard to the dependent variables, there were 6 studies for depression (20.0%), 8 for cognitive functions (26.7%), and 16 for other variables, including daily life performance ability, stress, coordination ability, self-integration, and so on (53.1%).

### 3.2. Overall Effect Size Analysis

Table 6 shows the results of analyzing the overall average effect size on the therapeutic recreation programs effects on the elderly.

**Figure 1:**
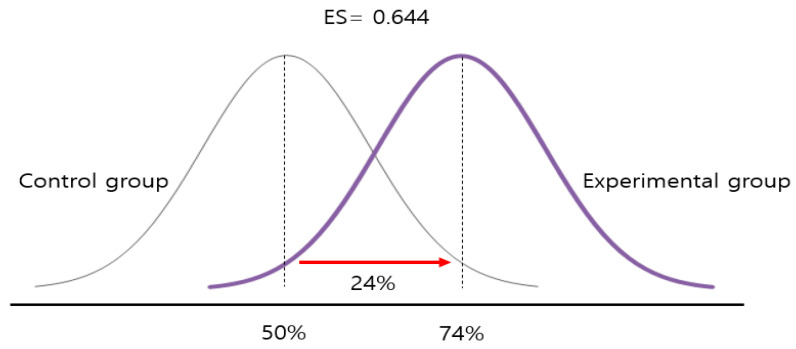
Interpretation of overall effect size.

First, with regard to the results of the homogeneity test, it was found that the effect size extracted from the research subject for the meta-analysis was heterogeneous (Q = 48.079, *p* < 0.001, $I^{2}$ = 70.882). Therefore, in this study, the overall effect size was analyzed with a random effect model. The overall effect size by the wireless effect model was 0.664, which was significant in the 95% confidence interval. The overall average effect size corresponds to the median effect size based on Cohen’s [24] proposed effect size interpretation criteria. In addition, when the overall effect size is analyzed through the Percentiles of non-overlap, assuming the control groups percentile is 50.0% in the normal distribution curve, the percentile of the experimental group is 74.0%, showing that the effects of the therapeutic recreational program was more effective than for the non-experimental group by 24%, as shown in Figure 1.

**Figure 2:**
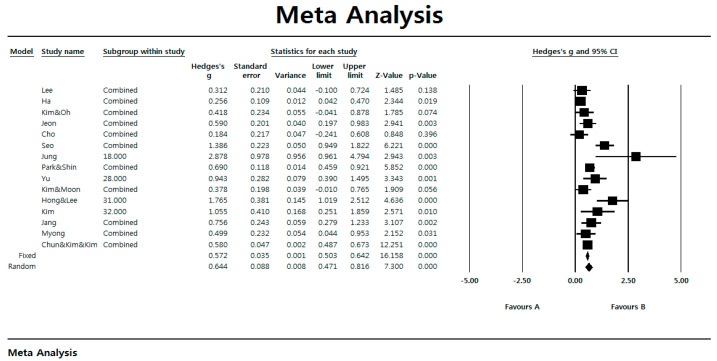
Forest plot.

Figure 2 shows is a forest plot showing the summary statistics of the research subject to be analyzed.

**Figure 3:**
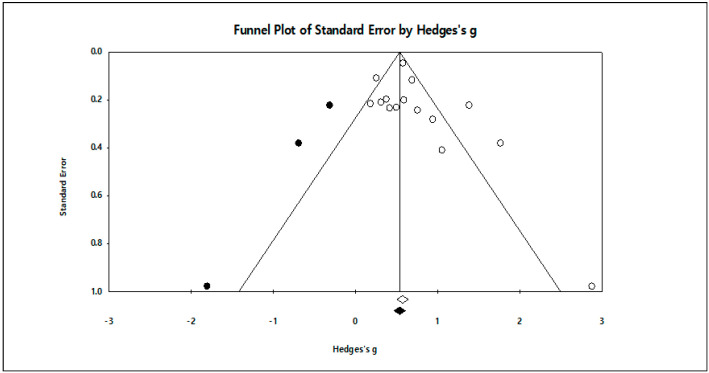
Funnel plot.

Meanwhile, in order to secure the validity of the meta-analysis results, a publication bias test was conducted. First, as a result of visually examining the degree of deflection through a funnel plot with the x-axis as the effect size (Hedges’) and the y-axis as the standard error, as shown in Figure 3, there are no signs of excessive deflection. 

In addition, as a result of examining the possibility of publication bias trough the estimate and subtraction method (trim and fill), as shown in Table 7, three studies showed correction values, and after adding these threes studies the effect size was 0.530, which showed that the effect size had decreased somewhat after correction, but the change was not enough to be significant (see Figure 3). 

### 3.3. Effect Size Analysis by Dependent Variable

Table 8 shows the effect size analysis results for the dependent variable. As a result of analyzing the effect size by classifying the dependent variables into social, emotional, physical, and cognitive domains, the social and emotional domains were 0.739, the physical domain was 0.548, and the cognitive domain 0.485, while the effect size of each domain was 95%, which was found to be significant in the confidence interval. According to the effect size analysis criteria proposed by Cohen [24], the effect size for each dependent variable showed an intermediate effect size in all areas. Additionally, the Percentiles of non-overlap for the effect size was 77.0% for the emotional areas, 70.8% for the physical areas, and 68.6% for the cognitive area.

### 3.4. Adjustment Effect Analysis

As a result of the homogeneity test for the overall average effect size, the effect size extracted from the research included in the analysis object appears to be heterogeneous (70.882%), so in order to identify the cause of heterogeneity, sub-group analysis and meta-regression analysis were performed on the main variables suggested in the research subject. The results of the adjustment effect analysis are shown in Table 9 and Table 10. 

The effect size for the study subjects were 0.691 for the healthy elderly and 0.574 for the elderly with any form of disease, each of which was found to be significant in the 95% confidence interval. The difference in effect size according to the study subjects, however, was not statistically significant (=0.819, df = 1, *p* = 0.366). The effect size for each gender grouping resulted in 0.696 for the female group and 0.578 for the mixed gender group, with each effect size showing significance in the 95% confidence interval. The difference in effect size according to gender was not statistically significant (=0.685, df = 1, *p* = 0.408). The effect size for the number or participants in the program was 0.706 for 10 or less, 0.562 for 11 or more and each effect size was significant in the 95% confidence interval. The difference in effect size according to the number of participants in the program was not statistically significant (=1.225, df = 1, *p* = 0.268). The effect size for the program frequency was 0.736 for once a week and 0.601 for twice a week; each effect size was significant in the 95% confidence interval. The effect size difference according to the program frequency was not of significance to the statistics (=0.595, df = 1, *p* = 0.440). The effect size for the application period showed a tendency that the effect size increased as the period increased, and was statistically significant (*p* < 0.05). The effect size for the tie per program session tended to increase as the time increased and was statistically significant (*p* < 0.05). 

## 4. Discussion

In this study, meta-analysis was performed to comprehensively analyze the effects of a therapeutic recreation program on the elderly. The main results derived from this study are as follows. 

First, the research status by year was found to have a relatively large proportion of research before 2010. These results indicate that the attempts to verify the effectiveness of programs focusing on just one component have increased in current studies compared to the previous studies focusing on verifying the effectiveness of a therapeutic recreation program by examining various factors, including types of body, play, reading, art, music, gardening, and laughter activities. As a result of examining the genders of the study participants, no studies verifying the effectiveness of the program were conducted for only the male elderly. In order to discuss the possibilities of a more generalized effectiveness of the therapeutic recreation program, it is necessary to conduct more research whilst considering better gender balance in the study participants. It has been verified through analysis that there are a higher number of small groups (10 or less) that have been used for the verification of the effectiveness of the program. Sufficient sample size reduces the likelihood of type 2 errors to occur in experimental studies and can be the base for a proper interpretation and application of the study results [28]. 

With this being said, it seems that efforts to conduct research by calculating the appropriate sample size according to the experimental design will be required. With regard to the frequency and time per program sessions, no studies, which examined the programs performed more than 3 times per week, were found, and the studies, which examined the programs performed over 60 min per session, were confirmed to be relatively lower in proportion. It is thought that the frequency and time of the programs were adjusted considering the characteristics of the elderly participants. Concerning the duration, frequency, and time of the program, as well as the participants’ gender, some studies did not provide the information related to them. Failure to provide information on the characteristics of the programs may show lack in validity of the study results. Therefore, it is necessary to describe in detail the contents and procedures of the research methods. 

Second, through the analysis of the overall effect size, a significant effect on the therapeutic recreation program of the elderly was confirmed. These results were somewhat consistent with the results of the prior studies [29,30,31] that proved the effectiveness of the program for the elderly through the use of meta-analysis. This will serve as an objective base for more active use of therapeutic recreation as a means of promoting the health of the elderly. Meanwhile, the overall effect size of the therapeutic recreation programs for the elderly was confirmed to be of medium effect size (0.661). These results show a slight difference from the large effect size revealed in prior studies [30,31], which implies that a greater effect can be expected when a single recreation activity is applied with more focus compared to when multiple recreational activities are applied in a complex manner. 

Third, in the effect size analysis for the dependent variable, the physical area showed a relatively low effect size compared to the emotional areas. These results fall in line with previous studies [9,10,14,32] that support the reports that therapeutic recreation programs had no significant effect on the physical areas, such as daily living ability or cardiopulmonary function in the elderly. Considering that the participants were the elderly, it was carefully noted that the reason that there were no significant improvement in physical function could be related to the fact the programs were more based on rhythm games and gymnastics rather than intense physical exercise. 

Fourth, a significant change in the effect size according to the duration of the program was confirmed. This result implies that the duration of the program will bring upon different effect sizes, and suggests that it is necessary to consider the duration of the program significantly when creating a therapeutic recreation program for the elderly. In particular, the effect size seemed to increase as the duration of the program increased as well. In light of this, it seems that going with a longer duration of the therapeutic recreation program will benefit the elderly more within the field of physical education. Furthermore, significant changes in the effect size, according to the duration of the program were confirmed. This result, in turn, shows that the effects of the program are very dependent on the duration of the program, and that it is important to consider it when constructing the program. On another note, as the duration of the program increased, the effect size tended to increase as well. Therefore, in order to expect high effectiveness of the program, it is ideal to avoid shorter timed program. Finally, it should be mentioned that no significant difference in effect size was found in the participants’ disease status, gender, number of participants, and frequency. Therefore, the insignificant differences in effect size for each of these areas did not have a significant impact on the effectiveness of the program. However, there is a difference in the effect size for each area despite it being insignificant; thus, it is important to be cautious for the interpretation of the analysis results.

## 5. Conclusions and Suggestions

The purpose of this study was to suggest the development direction of future programs by examining the effectiveness of therapeutic recreation programs for the elderly and to identify the factors affecting the effectiveness for better results. For this, a meta-analysis was conducted on studies that verified the effectiveness of the therapeutic recreation program for the elderly in Korea. The following conclusions were drawn based on the main results for the average effect size, the effect size for each dependent variable, and the adjustment effect analysis according to the research problem set in this study. First, the therapeutic recreation program is effective in bringing positive changes to the elderly, and the level of the effect is considerable. Second, the program has a relatively high effect on the social/emotional domain followed by the physical domain and the cognitive domain. Third, the time and the duration of the program have an influence on the program effectiveness, and the higher the duration and time, the higher the effect. This study recognized the value of therapeutic recreation activities for the elderly and suggested a direction which can increase the effectiveness of the program. In addition, the status of prior studies that verified the effectiveness of the therapeutic recreation program for the elderly is diagnosed, and it is significant in that it suggested the direction of subsequent studies. 

Proposals for follow-up studies reflecting the limitations of this study are as follows. First, this study limited the research participants to the elderly in the process of selecting data for the meta-analysis. Considering that a large number of studies have been found in the data collection process, to which a therapeutic recreation program has been applied to adolescents, middle aged persons, and people with disabilities, in subsequent studies, it is crucial to conduct a meta-analysis study on the effectiveness of the therapeutic recreation program with a larger variety of participants. Second, the data subject to meta-analysis in this study was limited to domestic studies. In the follow-up studies, it is necessary to conduct meta-analysis research, including foreign research.

## Figures and Tables

**Table 1:** Lists of analyzed papers for meta-analysis.

Author (Year)	Publication Type	Title of Papers
Lee (2012)	Dissertation	A study on a therapeutic recreation program the decreasing depression and improving of cognition of the elderly with dementia in nursing home
Ha (2002)	Dissertation	The effects of therapeutic recreation on cognitive function, depression, basic activity of daily life (ADL) in demented old adults
Kim and Oh (2016)	Publication	Study on effect of therapeutic recreation program to dementia aging people’s stress and cognition functions
Jeon (2007)	Dissertation	The effects of therapeutic recreation program on depression, self-esteem in demented old adults
Cho (2005)	Dissertation	A case study on the effectiveness of medical care recreation affected to the old with dementia: Focused on the acknowledgment function and the ability of accomplishing daily life
Seo (2007)	Dissertation	An empirical study on reality effectiveness of improving cooperation activity of therapeutic recreation to the old dementia: Focusing on tradition game program
Jung (2003)	Dissertation	A case study on the effect of leisure activities on the depression of the disabled elderly: The case of therapeutic recreation
Park and Shin (2000)	Publication	The therapeutic recreation effect for improving the women elder’s physical fitness
Yu (2010)	Dissertation	The effect of a therapeutic recreation program through traditional plays on acceptant attitudes of death of old people who have a dementia
Kim and Moon (2003)	Publication	An effect of the therapeutic recreation program on elder’s leisure awareness
Hong and Lee (2008)	Publication	A study on the development and effects of an educational program for ego integrity of the elderly through therapeutic recreation
Kim (2002)	Dissertation	The diminishing effect of therapeutic recreation program on degrees of depression of the elders
Jang (2006)	Dissertation	Study on effect of therapeutic recreation of a woman aged on estrangement diminution
Myong (2002)	Dissertation	A study on the effect of therapeutic recreation on the function of the physically challenged elderly and its use: through adult daycare service cases
Chun et al. (2001)	Publication	A study on the effect of therapeutic recreation program on psychological emotion of old women people

**Table 2:** Example of the coding form.

Category
1. ID
2. Author
3. Title of paper
4. Publication year
5. Publication type
6. Participants
7. Gender of Participants
8. Number of Participants
9. Dependent variable
10. Program period
11. Program frequency
12. Hours per session
13. Information of statistics

**Table 3:** Interpretation of effect size.

Effect Size (d)	Interpretation
0.30≤	Small size
0.40–0.70	Medium size
0.80≥	Large size

**Table 4:** Interpretation of $I^{2}$.

$I^{2}$	Interpretation
25%	Low heterogeneity
50%	Moderate heterogeneity
75%	High heterogeneity

**Table 5:** Characteristics of analyzed papers.

Variables	Categories	*n* (%)
Publication year	2000–2009	12 (79.9)
	2010~	3 (20.1)
Publication type	Dissertation	10 (66.7)
	Publication	5 (33.3)
participants of program	Senior	6 (40.0)
	Senior with disease	9 (60.0)
Gender of Participants	Only women	7 (46.7)
	Women and men	7 (46.7)
	Not described	1 (6.7)
Number of Participants	≤10	10 (66.7)
	11–20	3 (20.0)
	>20	2 (13.4)
Program period	6 weeks	2 (13.3)
	8 weeks	3 (20.0)
	9 weeks	1 (6.7)
	11 weeks	1 (6.7)
	12 weeks	4 (26.7)
	16 weeks	1 (6.7)
	17 weeks	1 (6.7)
	Not described	2 (13.3)
Program frequency	Once a week	6 (40)
	Twice a week	7 (46.7)
	Not described	2 (13.3)
Hours per session	≤60 min	11 (73.3)
	>60 min	3 (20.0)
	Not described	1 (6.7)
Dependent variable	Depression	6 (20.0)
	cognitive function	8 (26.7)
	Other Variables	16 (53.1)

**Table 6:** Overall effect size.

Model	K	ES	95% CI	*U*_3_	Heterogeneity	
					Q	$I^{2}$
Fixed	15	0.572	0.503–0.642	71.6	48.079 ***	70.882
Random	15	0.644	0.471–0.816	74.0		

*** *p* < 0.001.

**Table 7:** Analysis of trim and fill.

Value	Studies Trimmed	ES
Observed values	-	0.644
Adjusted values	3	0.530

**Table 8:** Effect size by dependent variable.

Dependent Variable	K	ES	95% CI	*U*_3_
Social-emotional	21	0.739	0.567–0.911	77.0
Physical	14	0.548	0.313–0.783	70.8
Cognitive	8	0.485	0.186–0.784	68.6

**Table 9:** Sub-group analysis.

Sub-Group	K	ES	95% CI	U3	*Q^b^*	df	*p*
Senior	22	0.691	0.523–0.860	0.755	0.819	1	0.366
Senior with disease	21	0.574	0.382–0.765	0.717			
Only women	27	0.696	0.534–0.858	0.757	0.685	1	0.408
Women and men	14	0.578	0.350–0.807	0.718			
≤10 people	30	0.706	0.534–0.877	0.760	1.225	1	0.268
>11 people	13	0.562	0.373–0.750	0.713			
Once a week	15	0.736	0.463–1.009	0.769	0.595	1	0.440
Twice a week	21	0.601	0.393–0.808	0.726			

**Table 10:** Meta-regression analysis.

Period and Hour		Estimate	Standard Error	z	*p*
Program period	Slope	0.047	0.023	2.065	0.039
	Intercept	0.099	0.261	0.381	0.703
Hours per session	Slope	0.010	0.004	2.346	0.019
	Intercept	−0.057	0.292	−0.194	0.847
